# Cogitations and Vaticinations

**Published:** 1856-04

**Authors:** 


### ECLECTIC DEPARTMENT,

### AND SPIRIT OF THE MEDICAL PERIODICAL PRESS.

*To be taken "cum grano salis."*—Dissenting from many of "An Old Fogy's" conclusions, we give the article for its suggestive character and pithy style.—Ed. Buf. Med. Jour.

### *Cogitations and Vaticinations.* By An Old Fogy.

An honest physician is the noblest work of God. Medicine offers more temptations than any other profession or calling, to imposture, to quackery, to snobbishness; in a word, to dishonesty. He who resists all these temptations is an honest and conscientious man, *par excellence.*

Medicine is an arcanum. Even those who make it the study of a lifetime understand it but in part—see its principles and laws as through an obscure glass. To the great public, it is a sealed book, an unsolved problem; yet medicine concerns the highest interests of humanity. The Father of Lies uttered an incontrovertible truth, when he said, "yea, all that a man hath he will give for his life."

Because the people are ignorant in regard to medicine, they are superstitious in their estimate of its powers; they are superstitious in their estimates of the qualifications of its practitioners; they follow the dictates of common sense in regard to every other interest; they judge well of the best blacksmith, the best builder, the best steamboat pilot, the best engineer. They know enough to judge accurately in regard to commercial and political interests. They even show remarkable acumen in the discussion of metaphysics; but of medical men and medical science they are mainly ignorant. Men learned in all things else, are fools in medicine. Men to whom all things else in heaven or on earth appear familiar, seem but babes in the knowledge of medical men and things. Hence, the eminent jurist, the successful merchant, the distinguished politician, the eloquent divine, employ as their physician the ignorant and designing quack; they have an idea that there is such a thing as medical tact—that some persons are born to be doctors—that there is a virtue in the touch and the looks of those on whom the gods or some auspicious star have shed their benign influences. They believe that the seventh son is more fitted for medicine than the sixth son. They look on medical skill rather as an inspiration than as the result of patient observation and profound study—as a sort of witchcraft rather than as an ordinary science—as a gift of Heaven or of Hell, as the case may be, rather than as the acquisition of labor and thought.

The people are profoundly ignorant of medical science; hence, the ignorant pretender successfully competes with the honest man of science for their patronage and favor. But cannot the man of science vindicate his claim by an appeal to reason; can he not show by his knowledge of the human system, that he is the superior of the quack? Talk of physiology and pathology, of dyscrasies, of pseudo-plasmata, of cachexias, to the people! They will take you for a madman, and prefer Brandreth with his blood-purifying pills or Jayne with his never-failing expectorant. With them, medicine is a sort of theology wrapt in mystery, beyond the reach of the intellectual grasp.

Man is a creature of hope—he never dies. The *post-mortem* dream succeeds the day dream of life, and even when he is in his grave he hardly realizes that he is dead at last. He hopes on to the last. Life on earth, with its refreshing air and its bright suns, and stars, and flowers, and friends, is sweet, very sweet. The undying spirit within him gives him the feeling of immortality. He die! no; he will yet live—there are so many medicines—so many doctors! There is yet a remedy. Was not such an one, just with the same disease, cured by such a doctor, or such a nostrum? Why should he not be cured as well as others? The ignorance and the hope of mankind, these are the two great facts on which the vultures of charlatanry have feasted and fattened. These are the foundations on which have arisen the ducal palaces of therapeutical knaves. I pass over the Townsends, the Moffats, the Perkinses, and such like—nothing better was to be expected of them. I pass by homœopathy, and hydropathy, and electropathy—some of the followers of these *isms* may even be honest. I come directly to regular old school, scientific medicine, and I ask myself, are we of the legitimate profession guiltless? Can we say to suffering humanity, "'shake not thy grey locks at us,' for we have always acted in good faith?" Alas, I fear that we are not by any means immaculate. "It is easier for a camel to pass through the eye of a needle than for a rich man to enter the kingdom of heaven." I hope that honest doctors will not be found so rare as rich saints.

I doubt not that many may be found; but the ignorance and the hope of the afflicted—their prejudices and their whims, and the natural cupidity of man, are strong temptations to imposture. Why not give the patient a little medicine? he thinks that he needs it. If I do not prescribe a drug, he will apply to another. I will prescribe a *medicina mentis*, a medicine for the mind. Why not use the speculum vagina? the patient has heard of it, and thinks her case calls for that very instrument. I will use it every three days; it will do no harm, and its frequent use will justify a large fee. Though I can see only the os uteri, she will believe that I understand all the mysteries of her disease, when I have "looked through the speculum." The patient has a chronic or an acute dysentery—it will inspire him with a remarkable degree of confidence if the physician employ the speculum ani. He is dull of hearing—perhaps has been deaf for a quarter of a century—have at him twice a week, at any rate, with the speculum auris. He is blind—bring out the opthalmoscope, and look at the arteria centralis retinæ: he will believe that you saw to the centre of his brain. He has consumption—examine his sputa with the microscope, and tell him that you found a neucleolated cell, and that he is in no danger, as tubercle is aplastic. He is in the last stage of phthisis, his very throat is tuberculous—tell him that only the throat is diseased, and that you have a way of curing that, by applying the specific to the very spot affected—all that is necessary is the probang and a solution of caustic. Alas, poor human nature! thy love of life, thy credulity, thy faith, thy hope, are mines for the impostor, richer than those of California, Australia and Ophir. Thou art blameless, or at least sufferest the penance due to thy faults; but a deep damnation awaits him who would levy black mail on thee, on the verge of the grave.

I believe that no profession can boast of a larger proportion of honest, conscientious men than that of medicine. One thing, I think, is certain, the physician will never do what he thinks will injure his patient. If the devil were a doctor, I have no doubt he would do his best; but then the devil might do a great many things to impress the patient with the idea that his satanic majesty was more than an ordinary prescriber. He might do a great many *ad captandum* things. He might make a frequent use of many curious instruments that would do no harm except to aid in getting up a diabolical bill. I fear that this is sometimes done by some physicians. They may have the excuse that the patients require these things. They have heard of Banning's brace, and the speculum, and the horseshoe pessary; and if you do not use them you will be discharged, and some one called in who will use them. What are you to do under circumstances so trying? Might not the physician, just to please the patient, go through the formality of employing these various things? If the patient has an idea that a few mesmeric passes will do him good—might not the doctor make these passes? otherwise, the patient will call in Dr. Scott. I think the physician should never be influenced by such whims unless the patient is crazy, then he may operate on the mind as best he may. But, as a general rule, all dictation should be resisted. When the patient begins to dictate to the physician, the latter has lost already the confidence of the former in a great degree. Under such circumstances it were better to retire from the case than to commence with speculums, braces, pessaries and passes. But another will be called in who will do these things, and I shall lose my patron. Very well, so be it. If in your judgment these things are not of any service, they will not be apt to prove beneficial in the hands of others; and the patient will return to you after a time. Rely on your judgment, be supreme with your patient, and abide the result.

I believe that the pessary has been used much too often, and that the cases in which it might do good are very rare. It will go pretty much out of fashion.

I regard abdominal braces as an abomination. How can bracing the abdomen support the uterus?

I have no doubt that the speculum vagina has been used a thousand fold oftener than there was any necessity for it. What part of the womb can be seen by the speculum? only the little neck; the condition of which might be ascertained almost as well by the index finger. I admit the occasional utility of the instrument; but I do not believe that its employment is very often necessary. I do believe that the instrument has been greatly abused. A better time is coming for female modesty.

The probang has had its day. It cannot for ever be thrust down the throats of hoarse preachers and consumptives. The probang, I fear not to say, has done about as much harm as good, perhaps more. The probang, notwithstanding its elasticity and sponginess, is a doomed instrument. Green doctors may recommend it, but very few patients will be found green enough to submit to its use. In the name of common sense what good can cauterizing the larynx do in a tubercular consumption? E.en in an idiopathic laryngitis, would it not be likely to do as much harm as good? Would not the inhalation of a little impalpable powder of some astringent do as well? O, I think the inhalers, though as a general rule a humbug, are greatly superior to the probang. Apply the remedy right to the part affected! that's the idea—that takes with the deluded multitude; but, my deluded friend, the part diseased is not the part to which the remedy should be generally applied. The ministers of the gospel have suffered from the inhaler and the probang. Good for them! they are generally the patrons and apologists of medical humbug, and even of downright quackery.

I shall be regarded by some as an enemy of science, because I do not look upon every new notion as a progress, every innovation as a reform and an improvement. I am an old fogy, but still the friend of man, and of whatever ministers to his well-being. I am the enemy of pseudo-science. I would lift my voice against the abuse of any good thing. I am opposed to ranking things of subordinate importance higher than they deserve to be ranked. I have been accused of being the enemy of *pathological anatomy*—I am not. But *pathological anatomy*, the mere changes in the solids induced by disease is not so important, in a practical point of view, as it used to be fashionable to regard it. Pathological anatomy is but a description of the ruins of the edifice destroyed by the flames of disease; and an examination and classification of the cinders and charred timbers and ashes will not teach the best method of putting out the future fire. Pathological anatomy is mainly made up of the exuded plasma of the blood, variously organized and modified. Some physicians seem to be disposed to make a separate disease of every slight modification of the plasma. With them the grey tubercle is one disease, the yellow tubercle another disease. The tubercular infiltration is a third disease. The tubercles of the brain is a fourth, and so on. If the tubercle undergoes cretefaction or cornification, behold! it has been changed to a different disease. Every mutation of the morbid product is a distinct entity—a distinct species of disease, with the ultra pathological anatomist. I say, with Stokes, that pathological anatomy does not furnish a guide for therapeutics; and I predict that the day is not far distant when the pathological chemistry of the fluids will be looked to as the guide of the practitioner, rather than the pathological anatomy of the solids.

I am no great friend to specialities in medicine—to understand well a part, the whole must be understood. The old and time-honored division of our art into medicine and surgery, is a sufficient specialization, but the sub-divisions of latter times I look upon with distrust. I do not like to hear of eye doctors, and ear doctors, and lung doctors, and kidney doctors, and those who profess to treat only the diseases of the *sartorius muscle*, or the *tendo achilles*. What are we coming to? Shall we not have one doctor for the right eye and another for the left? one for the internal and another for the external ear? The times are progressive—so it is contended. A long time used to be required to ascend that delectable height where stands the fair temple of medical fame. In these days of the electric telegraph and railroads it is not so difficult. We now have medical associations, and schools, and conventions, in abundance, by which Broussais', and Rushes, and Andrals, are made on the shortest notice.

Long time ago, in the dark ages, or even later, the doctor was fortunate if in a half a score of years

"His name full six miles round the country ran;"

but now the ambitious aspirant can be known from Maine to California, by attending a medical convention. The price of knowledge, and books, and fame, is very reasonable in our favored age and country.—*St. Louis Med. and Surg. Journal.*

